# Effects of peppermint (*Mentha x piperita* L.) oil on cardiometabolic outcomes in patients with pre- and stage 1 hypertension: A placebo randomized controlled trial

**DOI:** 10.1371/journal.pone.0344538

**Published:** 2026-04-23

**Authors:** Jonathan Sinclair, Benjamin Sant, XuanYi Du, Gareth Shadwell, Stephanie Dillon, Bobbie Butters, Lindsay Bottoms

**Affiliations:** 1 Research Centre for Applied Sport, Physical Activity and Performance, School of Health, Social Work & Sport, University of Lancashire, Lancashire, United Kingdom; 2 School of Medicine & Dentistry, University of Lancashire, Lancashire, United Kingdom; 3 Centre for Research in Psychology and Sport Sciences, School of Life and Medical Sciences, University of Hertfordshire, Hertfordshire, United Kingdom; Central Food Technological Research Institute CSIR, INDIA

## Abstract

Hypertension represents the predominant risk factor for cardiovascular disease morbidity and mortality; with significant healthcare utilization and expenditure. Pharmaceutical management is habitually adopted; although its long-term effectiveness remains ambiguous, and accompanying adverse effects are disquieting. Peppermint, which is rich in menthol and flavonoids, may exert potential benefits relevant to hypertension. This trial aimed to explore the effects of twice-daily peppermint oil supplementation in individuals with pre- and stage 1 hypertension. A 20 day, parallel randomized, placebo-controlled trial was adopted (NCT05561543). 40 individuals with pre- and stage 1 hypertension were randomly assigned to receive 100 μL per day of either peppermint oil or peppermint-flavoured placebo. The primary trial outcome was the between-group difference in systolic blood pressure from baseline to 20 days. Secondary outcome measurements were the between-group differences in anthropometric, haematological, diastolic blood pressure/resting heart rate, psychological wellbeing, and sleep efficacy indices. Statistical analysis was conducted on an intention-to-treat basis using baseline-adjusted linear regression models comparing post intervention values between trial arms with the corresponding baseline value entered as a covariate; adjusted mean differences (*b*), 95% confidence intervals, and effect sizes (*d*) were calculated. In relation to the primary outcome, adjusted systolic blood pressure at 20 days was significantly lower (*b* = −8.48 mmHg, 95% CI = −14.24 to −2.73, *d* = −0.94) in the peppermint trial arm (baseline = 130.05 mmHg, 20 days = 121.97 mmHg) than in placebo (baseline = 130.93 mmHg, 20 days = 131.05 mmHg). Loss to follow-up (N = 1) and adverse events (N = 1) were low, both occurring in the peppermint arm, and compliance was very high in the peppermint (93.3%) trial arm. Given the substantial health and economic burden associated with hypertension worldwide, these findings suggest that twice-daily peppermint supplementation may represent a simple, low-cost, and well-tolerated strategy to support blood pressure reduction in this population.

Trial registration

ClinicalTrials.gov NCT05561543

## Introduction

Globally, hypertension is renowned as the leading risk factor for cardiovascular disease morbidity and mortality [1]. High blood pressure ranks first among modifiable risk factors attributable to cardiovascular disease aetiology, accounting for the largest proportion of coronary heart disease, heart failure, and stroke events [2]. It is associated with significant societal and economic consequences [3] and also mediates significant productivity loss from disability and premature death [4]. Thus, hypertension is one of the most consequential and remediable threats to the health of individuals and society.

Pharmaceutical intervention is the predominant treatment approach for hypertensive disease, and angiotensin-converting enzyme inhibitors, beta-blockers, calcium antagonists, and diuretics are the most commonly adopted approaches [5]. However, while these medicines are effective for the treatment of hypertension, their long-term comparative effectiveness in routine care remains an area of ongoing investigation, with some evidence indicating differences between drug classes [6]. In addition, long-term adherence can be suboptimal [7], in part because adverse effects and treatment burden may influence continued use [8]. These considerations, alongside overreliance of daily prescription medication and broader preference among some patients for non-pharmacological options, support continued evaluation of adjunctive approaches with favourable tolerability profiles for the management of cardiometabolic risk [9].

Improved dietary practices are the principal approach for the non-pharmaceutical prevention and management of hypertensive and cardiometabolic diseases [10]. Enhanced intake of fruits and vegetables has definitively been shown to improve hypertensive and cardiometabolic disease symptoms [11]. However, maintaining a habitual dietary pattern high in fruits and vegetables has been shown to be difficult to accomplish [12]; therefore, supplementation potentially represents a more appealing treatment and prevention modality.

Peppermint (*Mentha x piperita* L.) is a recurrent flowering plant that cultivates in western Europe and North America. Peppermint is a hybrid of both spearmint (*Mentha spicata* L.) and water mint (*Mentha aquatica* L.). The peppermint plant contains a diverse chemical profile, including menthol, flavonoids, menthone, and menthyl acetate [13]. Peppermint possesses a broad range of biological activities, including digestive, choleretic, carminative, antiseptic, antibacterial, antiviral, antispasmodic, antioxidant, anti-inflammatory, myorelaxant, expectorant, analgesic, tonic, and vasodilatory properties [13,14], and has importantly been shown through toxicology analyses to be safe for ingestion [15].

Importantly, owing specifically to its antioxidant, anti-inflammatory, and vasodilatory properties, there is growing speculation that peppermint ingestion may target the mechanisms central to hypertensive pathophysiology, and thus confer significant clinical benefits [16]. To date, only very limited studies have been undertaken exploring the influence of peppermint supplementation on cardiovascular outcomes, with Barbalho *et al.* [17] showing that twice daily supplementation of peppermint, mediated significant reductions in both low-density lipoproteins (LDL) cholesterol and systolic blood pressure. However, this investigation did not feature a control group, meaning that the improvements cannot be attributed conclusively to peppermint supplementation, as opposed to other external mechanisms. Importantly, in healthy individuals, Sinclair *et al.* [16] showed using a placebo randomized controlled trial, that twice daily peppermint supplement yielded significantly greater reductions in systolic blood pressure, triglycerides and state/ trait anxiety compared to placebo.

At the current time, there has yet to be any randomized placebo-controlled intervention studies, examining the efficacy of peppermint supplementation in hypertensive individuals. Therefore, with preliminary evidence in healthy individuals suggesting a positive effect of peppermint ingestion [16], further placebo-controlled investigations concerning its influence on outcomes pertinent to hypertension may be of both practical and clinical relevance.

The aim of this placebo randomized trial is to investigate the effects of 20 days of twice daily peppermint supplementation in individuals with pre- and stage 1 hypertension compared to placebo. The primary objective of this trial is to investigate the effects of peppermint supplementation on systolic blood pressure relative to placebo. Its secondary objectives are to determine whether peppermint supplementation impacts upon other risk factors for hypertensive and cardiometabolic disease.

In relation to the primary outcome, it was hypothesized that peppermint oil will mediate statistically significant reductions in systolic blood pressure compared to placebo. Furthermore, for the secondary outcomes, peppermint oil will produce improvements in other cardiometabolic health parameters compared to placebo.

## Materials and methods

### Study design and setting

**Fig 1:**
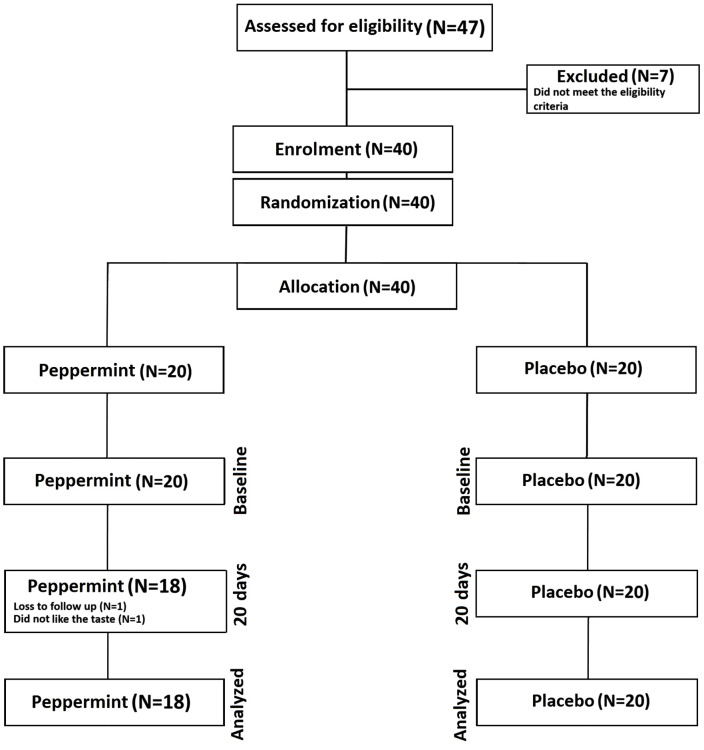
Consort diagram showing of participant flow throughout the study.

The comprehensive protocol for this study, detailing the study setting, CONSORT diagram, randomization process, recruitment strategy, and sample size calculation, has been previously published [18]. This study adheres to the latest guidelines for reporting parallel-group randomized trials [19] (S1). The University of Lancashire in the city of Preston in Lancashire, Northwest England, served as the location for the trial. In accordance with our previous trial, this research followed a 20 day parallel design, incorporating randomized allocation with a placebo control [16] (Fig 1). After screening for eligibility and enrolment, participants were randomized at the individual level, using a computer program (Random Allocation Software) to either a peppermint or placebo group. Screening included confirmation of eligibility and exclusion criteria via a structured health history review, including assessment for diagnoses suggestive of secondary hypertension and for major comorbidities that could influence blood pressure or participant safety. Indices, pertinent to hypertension, as described in detail below, were assessed at baseline and after 20 days (post-intervention). In agreement with previous trials involving hypertensive individuals, the primary outcome measure was the between-group difference in systolic blood pressure from baseline to post-intervention [9,20]. Secondary outcome measures were between-group differences in anthropometric, haematological, diastolic blood pressure/ resting heart rate, psychological wellbeing and sleep efficacy indices. All experimental visits took place in the morning and were undertaken in a ≥ 10-hour fasted state. Participants were also required to arrive hydrated and to avoid strenuous exercise, alcohol, and nutritional supplements 24 h and caffeine 12 h prior.

### Inclusion criteria

Eligibility criteria for this study required participants to meet the following conditions: (1) aged from 18–65 years; (2) fulfil the classification of pre- and stage 1 hypertension outlined by the American Heart Association [21], (3) not taking prescribed medicine for blood pressure management, (4) the ability to complete written questionnaires independently and (5) able to provide informed consent.

### Exclusion criteria

Exclusion criteria were (1) diagnosed diabetes mellitus; (2) known cardiovascular disease or clinically significant cardiovascular comorbidity, including coronary heart disease, symptomatic heart failure, clinically significant arrhythmia, or a history of stroke or transient ischaemic attack within the previous 6 months; (3) known or suspected secondary hypertension, including renal, renovascular, or endocrine causes; (4) known clinically significant renal impairment or severe hepatic disease; (5) evidence or history of severe hypertension related target organ damage requiring specialist management; (6) pregnant or lactating women; (7) allergy to peppermint; (8) habitual consumption of peppermint products; (9) regular consumption of antioxidant supplements; (10) body mass index larger than 40.0 kg/m²; (11) current enrolment in other clinical trials or use of other external therapies likely to influence outcomes; and (12) any condition likely to compromise informed consent, protocol compliance, or outcome assessment, including severe psychiatric illness, cognitive impairment, or active substance or alcohol misuse.

### Sample size

There has yet to be any investigation examining the efficacy of peppermint supplementation in hypertensive individuals. Therefore, a pragmatic a priori sample size calculation was undertaken based on our previous trial examining the effects of peppermint supplementation on systolic blood pressure (i.e., our primary trial outcome) in healthy individuals [16]. Considering an expected attrition rate of 10%, this revealed that 20 participants would be necessary in each trial arm, with a total N of 40, to achieve *α* = 5% and *β* = 0.80.

### Participants and recruitment

Recruitment for this project commenced on 01/12/2023 and continued until 07/07/2025 and data collection itself formally ended on 05/08/2025. Both males and females of diverse races and ethnicities, who live in Preston and its surrounding areas, were recruited. Recruiting materials were placed using public patient bulletin boards as well as using social media. Individuals expressing interest in participation were able to reach out to the research team for additional details about the study and to address any questions related to participation. Written informed consent was acquired from all participants.

### Ethical approval and trial registration

This study was granted ethical approval by the University of Lancashire HEALTH Ethics Committee (HEALTH 01074; S2-3), and all participants submitted written informed consent before participating, adhering to the principles stated in the Declaration of Helsinki. The trial was preregistered on clinicaltrials.gov (NCT05561543).

### Dietary intervention

After the conclusion of their baseline data collection session, participants were provided with either pure peppermint oil (Piping Rock Health, UK) or placebo. Participants randomized to the peppermint arm were required to consume 50 µL of supplement diluted into 100 mL of water twice daily: once in the morning and again in the evening. This dose was selected based on our previous placebo randomized trial in healthy individuals using the identical dose and supplementation schedule, which demonstrated a significant reduction in systolic blood pressure with no reported adverse effects or dropouts and high compliance (90.03%) in the peppermint trial arm [16]. The placebo condition involved the consumption of a peppermint-flavored cordial (Schweppes, Schweppes Geneva) in the same quantity and manner as the peppermint group, without the presence of peppermint oil, menthol, or peppermint-derived constituents listed on the ingredient declaration. The placebo cordial was selected based on its ingredient declaration, and this approach to placebo preparation has been shown in previous trials to provide an effective blinding strategy [16,22]. To ensure effective blinding, identical opaque 15 mL dropper bottles without any labels were supplied to participants in both the placebo and peppermint trial groups, with the only difference being the solution, i.e., placebo or peppermint that they contained. Additionally, all supplements were prepared by an independent researcher to maintain blinding.

Both the peppermint oil and peppermint-flavored cordial utilized in this trial are commercially available, food-grade products that are approved for human consumption. Pure peppermint oil is marketed as a dietary supplement and listed as a Generally Recognized As Safe (GRAS) substance by the U.S. Food and Drug Administration (21 CFR §182.20). The peppermint-flavoured cordial is a commercially available beverage produced in compliance with UK and EU food safety regulations, approved for general sale and consumption under the UK Food Safety Act 1990 and the Food Information Regulations 2014. Furthermore, peppermint flavourings contained within the cordial are permitted under EU Regulation No. 1334/2008 on flavourings and food ingredients with flavouring properties. The selected dose in the present trial was derived from our previous human study [16], which demonstrated significant improvements in cardiovascular outcomes without adverse events and with high participant compliance, supporting both the tolerability and safety of the intervention.

Throughout the study, the participants were encouraged to maintain their habitual diet and exercise routines; and asked to refrain from consuming any other peppermint supplements. Participants were also asked to keep a 4-day diet diary prior to the baseline assessment and before the follow-up examination at the end of the 20 day treatment period [9,20]. This ensured that there were no differences in dietary patterns between groups and that participants had not made significant changes to their nutritional approach that could influence the study outcomes. Diet diaries were analyzed using WinDiets Nutritional Analysis Software Suite Version 1.0 (Robert Gordon University, Aberdeen, UK), allowing daily energy intake, fat, saturated fatty acids, protein, carbohydrate, sugars, fibre, alcohol, vitamin A, thiamine, riboflavin, niacin, vitamin B6, vitamin B12, folate, vitamin C, vitamin D, vitamin E, calcium, salt, iron, zinc, and selenium to be examined.

For their post-intervention data collection session, all participants were asked to return any unused supplementation/ placebo to the laboratory in order to determine the % compliance in each trial arm. Furthermore, in order to examine blinding efficacy, each participant was asked which trial arm that they felt that they had been allocated to at the conclusion of their post-intervention data collection session. In both groups loss to follow up was monitored, as were any adverse events.

### Data collection

#### Blood pressure and resting heart rate.

Blood pressure and resting heart rate measurements were undertaken in an upright seated position. Peripheral measures of systolic and diastolic blood pressure and resting heart rate were measured via a non-invasive, automated blood pressure monitor (OMRON M2, Kyoto, Japan), adhering to the recommendations specified by the European Society of Hypertension [23]. Three readings were undertaken, each separated by a period of 1 min [24], and the mean of the last 2 readings used for analysis.

#### Anthropometric measurements.

Anthropometric measures of mass (kg) and stature (m) (without footwear) were used to calculate BMI (kg/m^2^). Stature was measured using a stadiometer (Seca, Hamburg, Germany) and mass measured using weighing scales (Seca 875, Hamburg, Germany). In addition, body composition was examined using a phase-sensitive multifrequency bioelectrical impedance analysis device (Seca mBCA 515, Hamburg, Germany) [25], allowing percentage body fat (%) and fat mass (kg) to be quantified. Finally, waist circumference was measured at the midway point between the inferior margin of the last rib and the iliac crest and hip circumference around the pelvis at the point of maximum protrusion of the buttocks, without compressing the soft tissues [26]; allowing the waist-to-hip ratio to be quantified.

#### Haematological testing.

Capillary blood samples were collected by finger-prick using a disposable lancet after cleaning with a 70% ethanol wipe. Capillary triglyceride, total cholesterol and glucose levels (mmol/L) were immediately obtained using three handheld analyzers (MulticareIn, Multicare Medical, USA). From these outcomes’ LDL cholesterol (mmol/L) was firstly quantified using the Anandarja *et al.* [27] formula using total cholesterol and triglycerides as inputs. In addition, HDL cholesterol (mmol/L) was also calculated by re-arranging the Chen *et al.* [28] equation to make HDL the product of the formulae. Both of these approaches have been shown to have excellent similarity to their associated lipoprotein values examined using immunoassay techniques r = 0.948–0.970 [28,29]. The ratios between total and HDL cholesterol and between LDL and HDL cholesterol levels were determined in accordance with Millán *et al.* [29]. Finally, the triglycerides and glucose (TyG index) was calculated as the natural logarithm of the product of plasma glucose and triglycerides divided by two [30].

#### Questionnaires.

Sleep quality has been shown to be diminished in patients with hypertension and cardiometabolic disease [31], and supplementation of peppermint has been demonstrated to enhance sleep quality [32]. Therefore, general sleep quality was examined using the Pittsburgh sleep quality index (PSQI) [33], daytime sleepiness using the Epworth Sleepiness Scale [34] and symptoms of insomnolence via the Insomnia Severity Index [35]. These questionnaires were utilized cooperatively to provide a collective representation of sleep efficacy. The Pittsburgh sleep quality index measure consists of 19 individual items, creating 7 components (subjective sleep quality, sleep latency, sleep duration, sleep efficiency, sleep disturbance, use of sleep medication, and daytime dysfunction) that produce a global score ranging from 0 to 21, with lower scores denoting a healthier sleep quality. The Epworth Sleepiness Scale consists of a list of eight scenarios in which tendency to become sleepy is rated on a scale of 0–3. The total score is the sum of these responses and ranges from 0 to 24, with higher scores indicating increased sleepiness. The Insomnia Severity Index features seven questions in which sleep difficulty is rated on a scale of 0–4. The total score is the sum of these responses and ranges from 0 to 28, with higher scores indicating greater sleep difficulty.

Because psychological wellbeing is lower in those with hypertension and cardiometabolic disease [36], general psychological wellbeing was examined using the COOP WONCA questionnaire [37], depressive symptoms using the Beck Depression Inventory [38] and state/ trait anxiety with the State Trait Anxiety Inventory (STAI) [39]. Once again, these scales were utilized conjunctively to provide a collective depiction of psychological wellbeing. The COOP WONCA questionnaire comprises six scales (physical fitness, feelings, daily activities, social activities, change in health and over-all health) designed to measure functional health status on a scale ranging from 1 to 5. The final score is the mean of the six scales, with a higher score indicating reduced functional health. The Beck Depression Inventory is a 21-item questionnaire in which depressive symptoms are rated on a scale of 0–3. The total score is the sum of these responses and ranges from 0 to 63, with higher scores indicating greater depression. Finally, the State-Trait Anxiety Inventory uses 20 items to assess trait anxiety and 20 to examine state anxiety, rated on a scale of 0–4. The total score for both trait anxiety and state anxiety is the sum of these responses for each component and scores range from 20 to 80, with higher scores denoting greater anxiety.

### Statistical analysis

Baseline demographic and clinical characteristics were presented descriptively for each trial arm. In accordance with CONSORT guidance, formal significance testing of baseline differences was not performed, and any observed differences were interpreted with reference to their prognostic relevance and the magnitude of any chance imbalance [19]. Continuous variables are expressed as means accompanied by their respective standard deviations, while categorical variables are reported as percentages (%) or frequencies (N). Comparisons of compliance levels (%) between trial arms were performed using linear regression models with trial arm included as a fixed factor.

All analyses of the intervention-based data adhered to an intention-to-treat approach. In accordance with our previously published trial protocol [18], treatment effects for all continuous outcome measures were estimated as between-trial-arm differences at 20 days, with adjustment for the corresponding baseline value of the same outcome. Accordingly, post-intervention values at 20 days were analyzed using linear regression models with trial arm included as a fixed factor and the corresponding baseline value entered as a covariate, an approach recommended for randomized controlled trials with baseline and follow-up continuous outcomes [40,41]. No additional baseline demographic or clinical characteristics were included as covariates in the primary models. For these analyses, the adjusted mean difference between trial arms at 20 days (*b*), 95% confidence intervals of the difference, and associated p-values are presented. Effect sizes were calculated as semi-standardised adjusted mean differences (*d*) by dividing *b* by the residual standard deviation from the fitted model [42]. Effect size values are interpreted as 0.2 = small, 0.5 = medium, and 0.8 = large [43].

The efficacy of blinding was assessed using a one-way chi-square (*Χ*^2^) goodness-of-fit test. Two-way Pearson chi-square tests of independence were applied for bivariate cross-tabulation analyses between trial arms. These analyses assessed the number of participants lost to follow-up and the incidence of adverse events in each group. Chi-square analyses were calculated using Monte-Carlo simulation to determine probability values. Missingness was limited to the 20 day post-intervention outcome values of two participants in the peppermint trial arm who did not complete the follow-up assessment; no baseline variables were missing. To preserve the intention-to-treat analysis set, missing 20 day outcome values were imputed using a fully conditional specification approach [44]. As the incomplete variables were continuous post-intervention outcomes, the imputation models were specified for continuous variables and were informed by treatment allocation and the corresponding baseline value of each outcome. All statistical analyses were performed using SPSS v29 (IBM Inc., SPSS, Chicago, IL, USA), and statistical significance was considered at the p ≤ 0.05 level.

## Results

### Baseline demographic, anthropometric, and health information

Baseline characteristics of participants are presented in Table 1. Baseline systolic blood pressure, the characteristic of greatest prognostic relevance to the primary outcome, was similar between the placebo and peppermint trial arms.

**Table 1:** Participant characteristics.

Sex	Total		Placebo		Peppermint	
**Mean**	*SD*	**Mean**	*SD*	**Mean**	*SD*
Male = 62.5%		Male = 60%		Male = 65%	
Female = 37.5%		Female = 40%		Female = 35%	
Age (yrs)	34.85	14.79	35.50	13.99	34.20	15.89
Mass (kg)	79.35	9.96	82.85	9.42	75.85	9.44
Stature (cm)	174.13	10.68	175.77	11.80	172.49	9.44
BMI (kg/m^2^)	26.23	3.17	26.99	3.81	25.46	2.21
Smoking status	Yes = 2.5%		Yes = 0%		Yes = 5%	
	No = 90%		No = 85%		No = 95%	
	Previous = 7.5%		Previous = 15%		Previous = 0%	
Marital status	Married/ Civil partnership = 47.5%		Married/ Civil partnership = 40.0%		Married/ Civil partnership = 55.0%	
	Divorced = 7.5%		Divorced = 5.0%		Divorced = 10.0%	
	Single = 45%		Single = 55.0%		Single = 35.0%	
Children	0 = 45.0%		0 = 55.0%		0 = 35.0%	
	1 = 12.5%		1 = 10.0%		1 = 15.0%	
	2 = 32.5%		2 = 30.0%		2 = 35.0%	
	3 = 7.5%		3 = 0%		3 = 15.0%	
	4 = 2.5%		4 = 5.0%		4 = 0%	
Ethnicity	Caucasian = 80.0%		Caucasian = 90.0%		Caucasian = 70.0%	
	Asian = 12.5%		Asian = 10.0%		Asian = 15.0%	
	Black = 2.5%		Black = 0%		Black = 5.0%	
	Mixed = 5.0%		Mixed = 0%		Mixed = 10.0%	
Alcohol (units/ week)	5.04	5.24	5.70	5.11	4.38	5.41
Education	High-school = 15.0%		High-school = 10.0%		High-school = 20.0%	
	College = 20.0%		College = 25.0%		College = 15.0%	
	Bachelors = 47.5%		Bachelors = 45.0%		Bachelors = 50.0%	
	Postgraduate = 15.0%		Postgraduate = 15.0%		Postgraduate = 15.0%	
	Doctoral = 2.5%		Doctoral = 5.0%		Doctoral = 0%	

*Notes: Continuous variables are presented as mean and SD; categorical variables are presented as percentages (Abbreviations: BMI = body mass index)*

### Compliance, loss to follow up, and adverse events

Total trial completion numbers in each group were peppermint N = 18 and placebo N = 20, with loss to follow-up (N = 1) and an adverse event (N = 1) occurring in the peppermint arm (Fig 1). The adverse event was minor and caused by the participant’s dislike of the taste of peppermint. The chi-square tests were non-significant, indicating that there were no statistically significant differences between trial arms in either loss to follow-up (p = 0.151) or adverse events (p = 0.311). There was no statistically significant difference (p = 0.565) in compliance between the peppermint (93.3%) and placebo (92.2%) trial arms.

### Blinding efficacy

Of the 38 participants that completed the trial, 47.4% (N = 18) correctly identified their designated trial arm, the Chi-squared test was non-significant (p = 0.746) indicating that an effective blinding strategy was adopted.

### Blood pressure and resting heart rate

Adjusted post-intervention systolic blood pressure (*b* = −8.48 mmHg, 95% CI = −14.24 to −2.73, p = 0.005, *d* = −0.94), diastolic blood pressure (*b* = −4.57 mmHg, 95% CI = −8.98 to −0.15, p = 0.043, *d* = −0.66), and resting heart rate (*b* = −8.92 beats/min, 95% CI = −17.43 to −0.40, p = 0.041, *d* = −0.72) at 20 days were significantly lower in the peppermint arm compared to placebo after adjustment for baseline values (Table 2).

**Table 2:** Blood pressure, anthropometric, haematological, and questionnaire measurements (Mean and SD) as a function of each trial arm.

	Placebo				Peppermint				*b*	95% CI		P-value	*d*
	**Baseline**		**20 days**		**Baseline**		**20 days**	
	**Mean**	*SD*	**Mean**	*SD*	**Mean**	*SD*	**Mean**	*SD*	**Lower**	**Upper**
Systolic BP (mmHg)	130.93	13.37	131.05	12.89	130.05	7.80	121.97	10.17	**−8.48**	**−14.24**	**−2.73**	**0.005**	**−0.94**
Diastolic BP (mmHg)	83.20	11.09	83.05	11.17	83.25	9.18	78.52	9.33	**−4.57**	**−8.98**	**−0.15**	**0.043**	**−0.66**
Resting heart rate (beats/min)	63.55	9.23	66.90	12.05	72.10	12.63	66.77	20.27	**−8.92**	**−17.43**	**−0.40**	**0.041**	**−0.72**
Mass (kg)	82.85	9.42	82.54	9.36	75.85	9.44	75.55	9.63	0.02	−0.70	0.74	0.954	0.02
Fat mass (kg)	21.72	8.86	21.89	9.01	18.79	7.41	18.87	7.10	−0.19	−1.46	1.08	0.763	−0.10
BMI (kg/m^2^)	26.99	3.81	26.89	3.75	25.46	2.21	25.36	2.35	0.01	−0.22	0.24	0.937	0.03
Body fat (%)	26.31	10.63	26.64	10.81	24.84	9.39	25.01	8.80	−0.22	−1.85	1.41	0.783	−0.09
Waist circumference (cm)	88.33	6.78	88.65	6.80	85.17	8.72	84.61	10.46	−0.60	−2.15	0.95	0.436	−0.25
Waist:hip ratio	0.85	0.06	0.85	0.06	0.84	0.08	0.84	0.10	−0.01	−0.03	0.01	0.407	−0.26
Total cholesterol (mmol/L)	4.16	1.00	3.92	0.89	4.05	0.72	3.66	0.57	−0.22	−0.64	0.20	0.299	−0.33
LDL (mmol/L)	2.47	0.91	2.26	0.75	2.35	0.65	2.10	0.54	−0.10	−0.46	0.25	0.559	−0.19
HDL (mmol/L)	1.27	0.35	1.26	0.37	1.29	0.20	1.20	0.13	−0.08	−0.19	0.04	0.193	−0.42
Total:HDL ratio	3.40	0.90	3.22	0.77	3.22	0.65	3.10	0.62	−0.04	−0.43	0.36	0.847	−0.06
LDL:HDL ratio	2.07	0.84	1.89	0.71	1.89	0.59	1.79	0.56	−0.02	−0.38	0.34	0.917	−0.03
Glucose (mmol/L)	5.11	1.50	5.08	1.39	4.84	1.74	4.87	1.31	−0.03	−0.56	0.50	0.921	−0.03
Triglycerides (mmol/L)	1.33	0.98	1.33	1.03	1.37	0.53	1.11	0.34	−0.25	−0.56	0.07	0.121	−0.50
TyG index	8.39	0.53	8.38	0.57	8.42	0.51	8.29	0.31	−0.10	−0.34	0.14	0.395	−0.27
Beck depression inventory	5.90	5.57	5.95	6.36	6.00	4.81	4.50	4.62	−1.55	−3.20	0.11	0.066	−0.60
COOP WONCA	1.98	0.46	1.88	0.56	1.78	0.40	1.69	0.48	−0.05	−0.33	0.24	0.732	−0.11
STAI state	33.45	8.36	33.20	10.89	31.65	8.56	31.07	11.02	−0.19	−4.13	3.75	0.924	−0.03
STAI trait	34.65	9.96	36.40	11.86	35.45	8.90	35.91	11.21	−1.15	−6.66	4.36	0.675	−0.13
PSQI	5.60	2.70	5.65	3.31	5.00	2.51	4.39	2.93	−0.74	−2.13	0.66	0.293	−0.34
Insomnia severity index	6.35	5.09	4.90	3.43	7.50	5.89	6.13	5.80	0.50	−1.61	2.61	0.633	0.15
Epworth sleepiness scale	5.05	2.96	4.40	2.84	7.15	3.41	6.51	2.69	0.57	−0.43	1.57	0.257	0.38

***Notes: b = adjusted mean difference at 20 days between the peppermint and placebo trial arms controlling for the corresponding baseline value; CI = confidence interval for b; p value = probability value for the adjusted between group difference at 20 days; d = semi standardised adjusted effect size. Negative b values denote lower adjusted values in the peppermint arm than in the placebo arm. Bold text indicates a statistically significant adjusted between group difference at 20 days. Abbreviations: BP = blood pressure, BMI = body mass index, LDL = low density lipoprotein, HDL = high density lipoprotein, TyG index = triglyceride glucose index, STAI = State Trait Anxiety Inventory, PSQI = Pittsburgh Sleep Quality Index.***

### Anthropometric measurements

Adjusted post intervention anthropometric measurements at 20 days did not differ significantly between the placebo and peppermint trial arms after controlling for baseline values (p = 0.407–0.954; Table 2).

### Haematological testing

Adjusted post intervention haematological parameters at 20 days did not differ significantly between trial arms after controlling for baseline values (p = 0.121–0.921; Table 2).

### Questionnaires

Adjusted post intervention questionnaire-based outcomes at 20 days did not differ significantly between trial arms after controlling for baseline values (p = 0.066–0.924; Table 2).

### Diet diaries

Across all dietary intake measures, adjusted post intervention values at 20 days were similar between the placebo and peppermint trial arms after controlling for baseline values, with no statistically significant between group differences observed (p = 0.241–0.992; Table 3)

**Table 3:** Dietary measurements (Mean and SD) as a function of each trial arm.

	Placebo				Peppermint				*b*	95% CI		P-value	*d*
**Baseline**		**20 days**		**Baseline**		**20 days**	
**Mean**	*SD*	**Mean**	*SD*	**Mean**	*SD*	**Mean**	*SD*	**Lower**	**Upper**
Energy intake (Kcal)	2027.64	722.45	1935.36	783.25	1771.00	459.41	1748.18	391.35	21.48	−330.81	373.76	0.900	0.06
Fat (g)	76.15	34.49	74.20	27.61	61.82	25.34	66.07	18.61	0.07	−14.98	15.13	0.992	0.00
Saturated fatty acids (g)	25.51	12.77	27.65	12.84	20.45	6.59	25.30	9.17	1.18	−6.98	9.34	0.765	0.13
Protein (g)	95.55	49.35	90.56	55.52	83.69	23.72	64.30	26.84	−17.25	−47.09	12.59	0.241	−0.52
Carbohydrate (g)	246.86	84.39	224.24	118.62	216.97	94.36	171.88	94.38	−27.07	−97.58	43.45	0.432	−0.35
Sugars (g)	109.83	57.51	103.55	74.75	97.05	39.92	76.89	43.29	−13.78	−46.36	18.80	0.387	−0.38
Fibre (g)	17.72	4.75	18.45	7.21	18.46	6.41	18.07	4.72	−0.80	−5.54	3.94	0.727	−0.15
Alcohol (mL)	7.22	9.54	4.95	8.08	5.82	9.16	6.72	14.19	2.01	−8.50	12.51	0.694	0.17
Vitamin A (ug)	639.45	454.55	829.55	698.48	561.00	345.46	583.18	332.48	−185.58	−598.61	227.45	0.359	−0.40
Thiamine (mg)	1.67	0.49	1.59	0.73	1.47	0.24	1.67	0.46	0.27	−0.20	0.73	0.250	0.52
Riboflavin (mg)	1.77	0.75	1.80	0.96	1.89	0.32	1.86	0.43	0.07	−0.62	0.75	0.838	0.09
Niacin (mg)	38.46	13.73	34.93	19.38	33.35	7.42	32.47	8.50	−0.21	−13.56	13.13	0.974	−0.01
Vitamin B6 (mg)	1.62	0.43	1.64	0.72	1.59	0.37	1.64	0.49	−0.01	−0.57	0.55	0.977	−0.01
Vitamin B12 (ug)	5.35	3.75	7.59	8.43	4.77	2.01	5.61	5.09	−1.90	−8.30	4.50	0.541	−0.27
Folate (ug)	233.45	71.14	224.36	98.88	243.55	56.00	285.45	143.26	50.29	−43.64	144.21	0.276	0.48
Vitamin C (mg)	79.20	49.20	66.91	35.68	95.65	77.62	84.38	43.48	14.13	−20.53	48.78	0.404	0.37
Vitamin D (ug)	2.30	1.70	3.48	2.33	3.17	1.65	3.33	2.92	0.02	−2.47	2.51	0.987	0.01
Vitamin E (mg)	8.70	3.91	7.39	3.00	7.47	3.43	7.88	3.24	1.01	−1.49	3.52	0.408	0.37
Calcium (mg)	929.18	374.20	925.55	428.67	1009.36	836.16	838.18	209.13	−101.75	−392.26	188.77	0.472	−0.31
Salt (g)	5.42	2.05	4.72	1.85	4.90	2.29	4.31	1.27	−0.22	−1.48	1.05	0.725	−0.15
Iron (mg)	11.88	4.60	11.30	6.16	13.18	7.81	9.85	3.33	−1.70	−6.11	2.70	0.428	−0.35
Zinc (mg)	12.33	10.73	11.40	11.18	10.47	5.00	8.12	2.19	−1.81	−6.00	2.38	0.378	−0.39
Selenium (ug)	63.91	24.47	52.18	29.43	64.82	32.21	41.82	17.37	−10.44	−32.47	11.60	0.334	−0.42

***Notes: b = adjusted mean difference at 20 days between the peppermint and placebo trial arms controlling for the corresponding baseline value; CI = confidence interval for b; p value = probability value for the adjusted between group difference at 20 days; d = semi standardised adjusted effect size.***

## Discussion

This trial aimed to evaluate the effects of a 20 day regimen of twice-daily peppermint supplementation on health indicators in individuals with pre- and stage 1 hypertension, relative to placebo. Notably, this study represents the first randomized controlled trial employing a parallel placebo-controlled design to investigate the impact of peppermint supplementation in this population. The primary objective was to examine the influence of peppermint supplementation on systolic blood pressure compared to placebo. Secondary objectives included assessing its effects on additional risk factors for hypertension and cardiometabolic disease.

In relation to the primary outcome, in agreement with our hypothesis and the findings of our previous trial in healthy individuals [16], adjusted systolic blood pressure at 20 days was significantly lower in the peppermint trial arm compared to placebo, with a large effect size. It is proposed that the observed benefits of peppermint supplementation were mediated by the presence of menthol. Menthol acts as an agonist for the transient receptor potential melastatin 8 (TRPM8) channels in vascular smooth muscle [45], with their activation subsequently triggering a vasodilatory effect. Specifically, the opening of vascular TRPM8 channels allows for the entry of calcium into the endothelium [46], which in turn stimulates nitric oxide production [47] and hyperpolarization of vascular smooth muscle cells [48]. Since arterial hypertension is the most common preventable risk factor for cardiometabolic disease [49], and the greatest single risk factor for global all-cause mortality [50], these findings have significant clinical implications. The results of this trial suggest that peppermint supplementation could be a valuable tool in the management of pre- and stage 1 hypertension.

In addition to the primary outcome, and in further support of our hypotheses, adjusted diastolic blood pressure and resting heart rate at 20 days were also significantly lower in the peppermint group compared to placebo. In addition to the aforementioned effects, it is proposed that the effects of peppermint in reducing the resting heart rate were also mediated as a function of menthol. In addition to the vascular effects described above, peppermint supplementation may also influence resting heart rate through autonomic nervous system modulation. Menthol has been shown to activate TRPM8 channels located on sensory neurons, which can alter autonomic balance by enhancing parasympathetic (vagal) activity and/or reducing sympathetic drive [51,52]. This shift in autonomic tone can reduce sinoatrial node firing rate, thereby lowering resting heart rate [52]. Importantly, epidemiological studies have shown that resting heart rate is an independent predictor of cardiovascular and all-cause mortality in both men and women with and without diagnosed cardiovascular disease [53,54]. Furthermore, epidemiological studies also suggest that reducing the resting heart rate is not only associated with decreased cardiovascular mortality but also with decreased all-cause mortality [55]. This observation provides further evidence that peppermint supplementation could be an effective tool in the management of cardiovascular disease.

Although significant reductions in systolic blood pressure, diastolic blood pressure, and resting heart rate were observed in the peppermint trial arm, it did not elicit statistically significant between-group differences in anthropometric, haematological, questionnaire-based, or dietary indices. It is ultimately beyond the scope of this trial and its experimental measures, to determine the mechanisms responsible for the lack of statistical differences in most secondary trial outcomes. However, the a priori sample size was determined to address the primary outcome and may therefore have provided limited statistical power to detect between-group differences in secondary trial measurements. In addition, the 20 day intervention period was designed to examine short-term responsiveness and may have been insufficient for detecting changes in outcomes that typically require longer exposure to manifest. Accordingly, larger trials of longer duration with follow-up are warranted to more definitively evaluate secondary endpoints and the sustainability of observed effects.

Overall, the current trial demonstrated a successful blinding strategy, a very low number of adverse events, good compliance, and a high retention rate in the peppermint group. Therefore, it can be concluded that peppermint is a safe, tolerable, and low cost (<£10 for 15 mL) modality for individuals with pre- and stage 1 hypertension, that can be easily incorporated into habitual dietary patterns. Notably, a significantly lower adjusted systolic blood pressure value at 20 days was observed in the peppermint trial arm, indicating that this supplement may represent an effective means of improving blood pressure in this population. However, it remains unclear whether these findings can be generalised to individuals in more advanced stages of hypertension or those with relevant comorbidities not examined in the present study. Further research is therefore warranted to establish whether the efficacy of peppermint observed in healthy individuals [16] and in the current cohort can be replicated in these populations. It is also notable that whilst other supplementary modalities such as Montmorency tart cherry and blueberry have also been shown to reduce systolic blood pressure and cardiometabolic risk factors [56,57], they necessitate the intake of increased sugar (≈15 g per 30 mL serving) and additional daily kilocalorie intake (≈80 kcal per 30 mL serving) [56,58]. In contrast, peppermint, administered in extremely small quantities relative to tart cherry or blueberry, may represent a more suitable option for supporting blood pressure control while aiding the maintenance of a healthy body weight.

As with any randomized controlled trial, this investigation is not without limitations. The a-priori sample size was determined to address the primary outcome and may therefore have provided limited statistical power to detect between-group differences in some secondary outcomes. Accordingly, null findings for secondary endpoints should be interpreted cautiously, and larger trials are warranted to more definitively evaluate these outcomes. A further limitation is the 20 day intervention period, which permits assessment of short-term blood pressure responsiveness but does not establish whether any effects are sustained. Given blood pressure variability and guidance that antihypertensive strategies should be evaluated over several months to establish maintenance of efficacy [59], longer trials with follow-up are required. Blood pressure outcomes were assessed using clinic style measurements obtained in a laboratory environment, which may not capture blood pressure throughout the day. Although more logistically and fiscally challenging, twenty-four-hour ambulatory blood pressure monitoring may be advantageous in nutritional interventions as it provides a more comprehensive depiction of systemic blood pressure across 24 hours and reduces the likelihood of white coat hypertensive readings [60]. Finally, while the present trial observed favourable effects of peppermint oil supplementation on blood pressure and selected cardiometabolic outcomes, it was not designed to elucidate the mechanistic basis for these changes. Menthol, a major constituent of peppermint oil, is a TRPM8 agonist and has been linked to vasodilatory effects via calcium dependent endothelial signalling and nitric oxide related pathways [45–48], but mechanistic indicators such as nitric oxide metabolites, endothelial function, and autonomic markers were not measured. Accordingly, mechanistic interpretation remains speculative, and future trials should incorporate such measures to evaluate underpinning pathways and optimise intervention delivery and clinical outcomes.

## Conclusion

The current placebo randomized controlled trial aimed to investigate the influence of 20 days of twice-daily peppermint supplementation on blood pressure and related health indicators in individuals with pre- and stage 1 hypertension, compared to placebo. The trial supported our primary hypothesis that peppermint supplementation would lead to a significant reduction in systolic blood pressure relative to placebo. Given the substantial health and economic burden associated with hypertension worldwide, these findings suggest that twice-daily peppermint supplementation may represent a simple, low-cost, and well-tolerated strategy to support blood pressure reduction in this population.

## Supporting information

S1 File CONSORT checklist. (DOC)

S2 File Research protocol. (PDF)

S3 File Institutional ethical approval. (PDF)
