# Supplementary material for: Effects of peppermint (Mentha x piperita L.) oil on cardiometabolic outcomes in patients with pre- and stage 1 hypertension: A placebo randomized controlled trial
Source: PLoS One. 2026 Apr 23;21(4):e0344538. doi: 10.1371/journal.pone.0344538 (PMC13105356; doi:10.1371/journal.pone.0344538)
Supplement: S2 File — (PDF) [file pone.0344538.s002.pdf]

# Ethics, Integrity and Governance Unit
# Ethics Application Form

**PLEASE NOTE THAT ONLY ELECTRONIC SUBMISSION IS ACCEPTED**

This application form is to be used to seek approval from one of the three University Ethics Review Panels (BAHSS; Science & Health). These Ethics Review Panels deal with all staff and postgraduate research student projects. Taught (undergraduate and MSc dissertation projects) will normally be dealt with via School/Faculty process / committee.

If you are unsure whether your activity requires ethical approval please complete a UCLan Ethics Checklist. If the proposed activity involves animals, you should not use this form. Please contact the Ethics, Integrity and Governance Unit within the Research and Enterprise Service – EthicsInfo@uclan.ac.uk – for further details.

Please refer to the guidance notes for help with completing the Ethics Form.

| | |
|---|---|
| If this application relates to project/phase which has previously been approved by one of the UCLan Ethics Review Panels, please supply the corresponding reference number(s) from your decision letter(s). ONLY REQUIRED FOR PHASED PROJECT | |
| **Previous Ethics Approval Ref No** | |
| **Student Registration No** | |

**1.0 Insurance Assessment Questions:** *Place a cross against the options that reflect your project (if applicable)*

| | | |
|---|---|---|
| Does any part of your research take place outside of the UK? | | ☐ |
| Does the research deliberately include participants from any of the following groups: | • pregnant women? | ☐ |
| | • children aged five or under? | ☐ |
| | • adults who lack the capacity to give informed consent? | ☐ |
| Does the research include medical intervention involving: | • investigating a medical device? | ☐ |
| | • a clinical trial? | ☐ |
| | • contraception? | ☐ |
| Does the research include more than 5000 participants? | | ☐ |
| Is the research to be carried out by other organisations where the University is required by contract to provide insurance cover for the research if it proceeds? | | ☐ |

**1.1 Project Type:**

What Research Theme does your project fall under? Lifelong Health and Wellbeing

Which Research Institute or Research Centre does your project align to? Centre: Applied Sport, Physical activity and Performance

| | | |
|---|---|---|
| ☒ Staff Research<br>☐ Commercial Project | ☐ Masters by Research<br>☐ MPhil Research<br>☐ PhD Research<br>☐ Professional Doctorate | ☐ Taught MSc/MA Research*<br>☐ Undergrad Research*<br>☐ Internship<br>*Please provide course name: |

**1.2 Principal Investigator / Director of Studies or Supervisor (if student project):**

| Name | School | Email |
|---|---|---|
| Dr Jonathan Sinclair | Sport and Health Sciences (AHRU, SENS & CASES) | jksinclair@uclan.ac.uk |

**1.3 Other / Co-Researchers / Student:**

| Name | School | Email |
|---|---|---|
| | Choose an item. | |
| | | |

**1.4 Project Title:**

Effects of oral peppermint supplementation on cardiometabolic parameters in participants with pre and stage 1 hypertension.

**1.5 Proposed Start Date:**

13/11/2023

**1.6 Proposed End Date:**

13/11/2025

**1.7 Is this project in receipt of any external funding?** (including donations of samples, equipment etc.)?

☒ Yes ☐ No

If Yes, please provide details of sources of the funding and what part it plays in the current proposal.

Peel Trust

**1.8 Project Description** (in layman's terms) including the aim(s) and justification of the project (max 300 words)

*Give a brief summary of the background, purpose and the possible benefits of the project. This should include a statement on the academic rationale, context of the activity and justification for conducting the project.*

Hypertension is the leading preventable risk factor for cardiovascular disease and all-cause mortality; responsible for >75000 annual deaths in the UK alone. In the UK, 31% of males and 26% of females experience this chronic condition, and in about 30% of individuals blood pressure remains uncontrolled. The population in the UK living with hypertension is now 14.4million, owing to ageing and growth of the population and exposure to lifestyle risk factors. Hypertension accounts for 12% of GP appointments, and is responsible for £2.1 billion of annual healthcare expenditure, representing the most expensive collective disease modality.

Peppermint (Mentha piperita L.) contains a diverse chemical profile, including menthol, flavonoids, menthone, and menthyl acetate. Due to its antioxidant, anti-inflammatory, and vasodilatory properties, peppermint may represent an innovative clinical modality targeting the mechanisms central to

hypertensive and cardiometabolic pathophysiology. Importantly, our recent trial showed that peppermint significantly improved systolic/diastolic blood pressure, blood lipids, health-related quality of life and psychological wellbeing in healthy individuals.

If peppermint shows positive results in those with hypertension, this may contribute to a change of strategy in the treatment of hypertension, patient wellbeing and financial sustainability of the NHS. Therefore, conducting a placebo randomized placebo-controlled trial to assess the influence of peppermint in pre and stage 1 hypertension is of clear scientific, practical and clinical relevance.

**The primary purpose of the proposed investigation is to test the ability of oral peppermint supplementation to improve cardiovascular parameters in individuals with pre and stage 1 hypertension.**

## 1.9 Methodology/ Protocol Please be specific

*Provide an outline of the proposed method, include details of sample numbers, source of samples, type of data collected, equipment required and any modifications thereof, etc. Please include:*

- *Aims and objectives:*
- *Hypotheses and/or research question(s) to be addressed:*
- *Study design, to include recruitment and sampling strategy, inclusion/exclusion criteria, sample size and justification*
- *Proposed method(s) of data analysis:*
- *Description of site(s) / facilities / equipment required:*

***The below protocol is identical to that approved previously (HEALTH 0016) the only differences are:***

1. ***The supplementation is peppermint and not cherry/ blueberry concentrate***
2. ***We are testing individuals with pre and stage 1 hypertension***
3. ***We are no longer doing the oxygen intake analysis using the Metalyzer gas analysis system***

20 participants per group, total N = 40 is the target for recruitment for this work, all of whom will be over the age of 18. Participants will be recruited from the general public. This sample size is based on our previous trial examining the effects of peppermint supplementation on systolic blood pressure (i.e. our primary trial outcome) in healthy individuals). Considering an expected attrition rate of 10%, this revealed that 20 participants would be necessary in each trial arm, with a total N of 40, to achieve $\alpha$ = 5% and $\beta$ = 0.80.

Participants will be scheduled to arrive at the Physiology laboratory (Darwin building 026) on a designated data collection day. After re-reading the information sheet they will be asked to complete a consent form and as per the information sheet will be advised to wear shorts and t-shirt.

Firstly, it will be necessary to determine whether volunteers meet the criteria for pre and stage 1 hypertension. This involves, participants having a systolic blood pressure in accordance with the American College of Cardiology and the American Heart Association guidelines. If participants meet the inclusion criteria, then they will be able to continue with the entirety of the below methodology and if not, then they will not be eligible for participation and will be provided with their tested blood pressure values and also have to opportunity to have their body fat % measured should they wish to do so.

To take part in this study participants to meet be (1) aged from 18-65 years; (2) fulfil the classification of pre-stage 1 hypertension outlined by the American Heart Association (21), (3) not taking prescribed medicine for blood pressure management, (4) the ability to complete written questionnaires independently and (5) able to provide informed consent.  Participants will be ineligible if they are (1) diagnosed with diabetes mellitus and coronary heart disease; (2) pregnant and lactating women; (3) allergy to peppermint, (4) habitual consumption of peppermint products, (5) regular consumption of

antioxidant supplements (5) body mass index (BMI) larger than 40.0 kg/m$^2$ and (6) current enrolment in other clinical trials of other external therapies.

Therefore, we will firstly measure participants blood pressure/ heart rate using a digital blood pressure monitor ( [illegible]. This will be done after they have sat quietly for a period of 5 minutes with the arm resting on the table.

For those meeting the above inclusion criteria we will then measure waist circumference at the height of the navel and hip circumference at the top of the femur bone using a tape measure.

Following this, body fat % will be measured using a specialist non-invasive machine. Specifically, body fat % will be quantified using bioelectric impedance (this is a completely non-invasive process which simply requires participants to stand with their feet on the base and hands on the hand rails

Furthermore, to meaasure blood based cardiometabolic parameters, capillary blood will be obtained by finger prick and examined using a hand held measuring device. This will allow triglycerides, blood glucose and total cholesterol to be obtained as well as LDL, HDL cholesterol, the ratios between total and HDL cholesterol and between LDL and HDL cholesterol, triglycerides and glucose index. The tester will wear

surgical gloves during the test and the testing area will be wiped down with antibacterial wipes before and after use

Finally, participants self-reported psychological wellbeing will be examined using the;

- Coop-Wonka chart
- Beck Depression Inventory
- State Trait Anxiety Inventory

Whereas sleep quality will be examined via the;

- Insomnia Severity Index
- Pittsburgh Sleep Quality Index
- Epworth Sleepiness Scale

Macro and Micro-nutrient intake will be measured using a 4 Day Diet Diary. As all the previously mentioned tests will be performed on the same day, this document will be emailed to the participants at least 1 week prior, which will allow the subjects to record everything they eat and drink during any consecutive 4-day period preceding the test date.

**(all questionnaires are attached).**

After completion of the first data collection procedure, participants will be randomly assigned to receive either 1. Peppermint supplementation, or 2. Placebo (peppermint cordial) – provided in identical dropper bottles. We will provide the supplementation and participants will be asked to consume (1 drop mixed into 100ml of water) once in the morning and once in the evening for twenty days. After twenty days, they will return to the laboratory and the above procedure will be repeated and the same measurements taken. Data collection will take around one hour for each session. Data from test 1 will be linked to test 2. Participants will return any un-used solution and will be asked which trial arm that they felt that they had been allocated to. If any are lost i.e. don't show up for their retest this will be monitored alongside and undesired effects.

In accordance with an identical randomized intervention study design by Sinclair et al, statistical comparisons of the change after 20-days between groups will be undertaken using linear mixed effects models with group (i.e. placebo or peppermint) modelled as a fixed factor and random intercepts by participants. Chi-squared tests will also be used to measure blinding and also undesired effects and participant loss.

**1.10 Has the quality of the project been assessed?** (select all that apply)

☐ Independent external review
☒ Internal review (e.g. involving colleagues, academic supervisor, School process)
☐ Research Programme Approval gained on Click here to enter a date. ***(Please note that RPA is a prerequisite for Research Degree Students and Professional Doctorates).***
☐ None

If other please give details

**1.11 Please provide details as to the storage and protection of your physical / electronic data for the next 7 years (or 10 years if funded by the EU or Medical Research Council)** – as per UCLan requirements – or whichever archive period is appropriate

The data will firstly be collected on a university surface pro/ surface laptop computer. Electronic files will be stored for a period of 7 years using a password protected file on the UCLan staff OneDrive system and processed using an institutional Surface Pro. All electronic data will be annonymized and

identifiable via a number only.

Paper data i.e. consent forms will be kept in a locked filing cabinet in the PI's office for the duration of the 7 year period.

**1.12 How is it intended the results of the study will be reported and disseminated?** (select all that apply)

☒ Peer reviewed journal – hard copy or online
☐ Internal report
☒ Conference presentation
☐ Other publication
☐ Written feedback to research participants
☐ Presentation to participants or relevant community groups
☐ Dissertation/Thesis
☐ Other

If other, please give details

**1.13 Will the activity involve any external organisation for which separate and specific approval is required?** (e.g. NHS; school; any criminal justice agencies including the Police, Crown Prosecution Service, Prison Service or Probation Service)?

☐ Yes ☒ No
*If **YES**, please refer to the guidance notes to determine what external approvals may be required.*

If Yes, please provided details of the external organisation and attach letter of approval

**1.14 The nature of this project is most appropriately described as research involving**: (more than one may apply)

☐ Behavioural observation
☒ Questionnaire(s) – please provide a copy of the questionnaire / survey
☐ Interview(s) – please provide a list of questions to be asked, or if semi-structured the topics
☐ Qualitative methodologies (e.g. focus groups) – please provide the questions/topics to be covered
☐ Psychological experiments
☐ Epidemiological studies
☐ Data linkage studies
☐ Psychiatric or clinical psychology studies
☒ Human physiological investigation(s)
☐ Biomechanical device(s)
☒ Human tissue(s)*
☐ Human genetic analysis
☐ A clinical trial of drug(s) or device(s)
☒ Lab-based experiment – please provide relevant COHSS / RA forms
☐ Archaeological excavation/fieldwork
☐ Re-analysis of archaeological finds/ancient artefacts
☐ Human remains analysis
☐ Lone working or travel to unfamiliar places (e.g. interviews in participants homes) – please provide relevant risk assessment form
☐ Other (please specify in the box below)

If 'Other' please provide details:

**1.15 Human Participants, Data or Material – the project will involve:**

Please select the appropriate box(es)

☒ Participants [proceed to next question 1.16]
☐ Data [proceed to question 1.30]
☐ Tissues /Fluids / DNA Samples [proceed to question 1.31] Please email EthicsInfo@uclan.ac.uk if a project involves this option.
☐ Remains [proceed to question 1.32]

**1.16 Will the participants be from any of the following groups: (tick as many as applicable)**

☒ Students or staff of this University (*Where staff or students of the university are being used please explain how this is not a convenience sampling in section 1.9)*
☐ Children/legal minors (anyone under the age of 18 years)
☐ Patients or clients of professionals
☐ Those with learning disability
☐ Those who are unconscious, severely ill, or have a terminal illness
☐ Those in emergency situations
☐ Those with mental illness (particularly if detained under Mental Health Legislation)
☐ People with dementia
☐ Prisoners
☐ Young Offenders
☐ Adults who are unable to consent for themselves
☐ Any other person whose capacity to consent may be compromised
☐ A member of an organisation where another individual may also need to give consent
☐ Those who could be considered to have a particularly dependent relationship with the investigator, e.g. those in care homes
☐ Other vulnerable groups (please list in box below)

If 'Other' please provide details

**1.16a Justify their inclusion**

*Ethics approval covers* ***all participants,*** *but attention must be given to those in a vulnerable category. Therefore, you need to fully justify their inclusion and give details of extra steps taken to assure their protection.*

Students and staff provide a convenient sample of individuals who may meet the inclusion criteria. Participating in the study will provide them with experience of seeing research conducted and of the equipment used.

It is important to note that whilst some participants may be UCLan staff/ students they will not be the only participants that are tested as part of data collection. Any other participants that respond to the advertisement poster (having read the information sheet) and meet the inclusion criteria will be eligible to take part.

**1.16b Is a DBS – Disclosure and Barring Service (formerly CRB – Criminal Records Bureau) check required?**

*Certain activities and/or groups of individuals require DBS (formerly CRB) clearance. If unclear, please seek advice.*

☐ Yes ☒ No

If Yes, please advise status of DBS clearance (e.g. gained; in process; etc.)

**1.16c All staff should be aware of UCLan's Policy and Procedures on Safeguarding and Prevent. Please confirm that, where relevant to your project, the appropriate training has been undertaken.**

*Please refer to UCLan Safeguarding Children, Young people and Vulnerable Adults Policy and Prevent guidance*

☐ Yes ☐ No ☒ N/A

If Yes, please give details of relevant training session – external or internal - and when (e.g. within last 3 years)

**1.17 Please indicate exactly how participants in the study will be (i) identified, (ii) approached and (iii) recruited**

If an advertisement and/or information sheet is being used, please attach

1. Participants will be required to meet the inclusion criteria outlined above. Those not meeting this criterion will not be selected for participation. Participants who do not meet the requirements for the study will be allowed to discuss the reasons with the experimenters if they wish.

2 & 3. The method of recruitment will be through advertisement of the project on social media (via Twitter @UCLanSNaCS); the advertisement posters are attached with this submission. Participants who express an interest in taking part who meet the requirements will be sent the information sheet to read. Provided that the study meets with their approval they will then be allowed to arrange a time to attend the lab for testing.

**1.18 Will consent be sought from the participants and how will this be obtained?**

If a written consent form is being used, please attach

Participants will be provided with an information sheet which explains the study and what will be required to them. They will then sign a consent form. See attached files.

**1.19 How long will the participants have to decide whether to take part in the research?**

Participants will have one week to decide whether to take part from the point of receiving the information sheet. Participants are free to withdraw at any point during the study, without having to give a reason and withdrawal from the study will not affect them in anyway. Participants will be able to withdraw their data from the study up until they have left the laboratory either on visit one and then separately on visit 2, after which withdrawal of their data from the visit will no longer be possible due to information being anonymized. If they choose to withdraw from the study before leaving the laboratory on either visit 1 or 2, no further data will be collected.

**1.20 What arrangements have been made for participants who might not adequately understand verbal explanations or written information, or who have special communication needs?**

*Give details of what arrangements have been made (e.g. translation, use of interpreters, etc.).*

No participants whom there may be issues providing consent will be recruited for this study. This is because of safety concerns. In a lab-based environment it is vital that the participants follow the instructions given by the experimenters clearly, given that none of the researchers are fluent in languages other than English or in sign language.

**1.21 Payment or incentives: Do you propose to pay or reward participants?**

☐ Yes ☒ No

If Yes, please provided details

**1.22 Will deception of the participant be necessary during the activity?**

| |
|---|
| ☒ Yes ☐ No |
| If Yes, please provide justification, and complete Question 1.28 |
| As a placebo controlled randomized trial, participants will be randomized to either placebo or peppermint groups and will not know what group they are assigned to. This is essential to the study design. |
| **1.23 Does your project involve the potential imbalance of power / authority / status, particularly those which might compromise a participant giving informed consent?** |
| ☒ Yes ☐ No |
| If Yes, please detail including how this will mitigated<br>*Describe the relationship and the steps to be taken by the investigator to ensure that participation is purely voluntary and not influenced by the relationship in any way.* |
| There is potential for students being taught by the PI to take part in the current investigation. However, the information sheet which is given to participants prior to them coming to the laboratory prior to data collection states very clearly that taking part will not influence their studies. |
| **1.24 Does the procedure involve <u>any</u> possible distress, discomfort, or harm (or offence)to participants or researchers?** (including physical, social, emotional, psychological and/or aims to shock / offend – e.g. Art)? |
| ☐ Yes ☒ No |
| If Yes, please explain<br>*Describe the potential for distress, discomfort, harm or offence and what measures are in place to protect the participants or researcher(s). Please consider all possible causes of distress carefully, including likely reaction to the subject matter, debriefing or participant upset.* |
| If adverse scores are present in any of the questionnaires, then participants will be advised to seek advice from their GP. |
| **1.25 Does the activity involve any information pertaining to illegal activities or materials or the disclosure thereof?** |
| ☐ Yes ☒ No |
| If Yes, please detail<br>*Describe involvement and explain what risk management procedures will be put in place.* |
| |
| **1.26 What mechanism is there for participants to withdraw from the investigation and how is this communicated to the participants?** |
| Participants may withdraw simply by communicating this to the researchers. The withdrawal process is also detailed in the information sheet. |
| **1.27 What are the potential benefits for the research?** |
| The research will inform future treatment practices for cardiovascular disease. |
| **1.28 Debriefing, support and/or feedback to participants** |
| *Describe any debriefing, support or feedback that participants will receive following the project and when.* |
| Participants will be given the hypothesis at the end of final data collection and told which condition that they were assigned to. This will be provided verbally. |
| **1.29 Will the project involve access to confidential information about people without their consent?** |
| ☐ Yes ☒ No |

| |
|---|
| If yes, please explain and justify<br>*State what information will be sought, from which organisations and the requirement for this information.* |
| |

| **1.30 Confidentiality/anonymity** - Will the activity involve: | | |
|---|---|---|
| | Yes | No |
| a. non-anonymisation of participants (i.e. researchers may or will know the identity of participants and be able to return responses)? | ☐ | ☒ |
| b. participants having the consented option of being identified in any publication arising from the research? | ☐ | ☒ |
| c. the use of personal data (i.e. anything that may identify them – e.g. institutional role – see DP checklist for further guidance)? | ☒ | ☐ |
| *If **yes to any** please attach completed Data Protection (DP) checklist* | | |

| |
|---|
| **1.31 Does the activity involve human tissue?‡** See **Human Tissue Act (HTA) Supplementary list of Materials** to check what is classified as human tissue. |
| ☒ Yes ☐ No |
| If no, please skip to question 1.32<br>If yes, please detail and answer questions 1.31a-c |
| |
| **1.31a Who will be sourcing the human tissue? (e.g. a tissue bank governed by its own HTA licence)** |
| N/A |
| **1.31b Will the human tissue be stored at UCLan? (please note restrictions on storage)** |
| ☐ Yes ☒ No |
| *If yes, please state how long and in what form - cellular or acellular (DNA extracted)*<br>*Please note – if human tissue is only kept for DNA extraction rendering it acellular the HTA storage regulations may not apply. If holding for DNA extraction, please state the length of time the tissue would be stored pre-extraction.* |
| Blood will be obtained using finger prick test and the sample will be analysed and disposed of immediately. There will be no storage.<br><br>Samples will be analyzed immediately and used for around 5 minutes which is the approximate time that the hand held analyzers take to give a reading. Samples will be destroyed by discarding the capillary sticks into a yellow waste disposal box which will be incinerated when full and no samples will be stored or kept overnight. |
| **1.31c Is the human tissue being used for an activity listed as a 'scheduled purpose' under Schedule 1 Parts 1 and 2 of the Human Tissue Act 2004?** *(click here to see list of HTA 'scheduled purpose' activities)* |
| ☐ Yes ☒ No |

| |
|---|
| **1.32 Does the project involve excavation and study of human remains?** |
| ☐ Yes ☒ No |
| If yes, please give details<br>*Discuss the provisions for examination of the remains and the management of any community/public concerns, legal requirement etc.* |
| |

| **1.33 Does the project involve working in a low or lower-middle income country as defined by the World Bank ?** |
|---|
| ☐ Yes ☒ No |
| *If yes, please explain how each article within the Global Code of Conduct for Research in Resource-Poor Settings has been considered with respect to your project:* *http://www.globalcodeofconduct.org/wp-content/uploads/2018/05/Global-Code-of-Conduct-Brochure.pdf*<br><br>**NOTE:** *If your research involves humans or animals, UCLan ethics/AWERB approval needs to be in place before application for local approval is made.* |
| |

‡ Until such time as the University gains its own HTA Research License, human tissue that is for a 'scheduled purpose' and not sourced from a BioBank or part of an NHS REC approved project can only be stored for a maximum of 5 days

## DECLARATION

This declaration needs to be signed by the Principal Investigator (PI), and the student where it relates to a student project (for research student projects PI is Director of Studies and for Taught or Undergrad project the PI is the Supervisor). Electronic submission of the form is required to EthicsInfo@uclan.ac.uk. Where available insert electronic signature – alternatively, provide an email in lieu from appropriate party.

| **Declaration of the:** |
| --- |
| Principal Investigator |
| **OR** |
| Director of Studies/Supervisor and Student Investigator |
| (please check as appropriate) |
| |

- The information in this form is accurate to the best of my knowledge and belief, and I take full responsibility for it.
- I have read and understand the University Ethical Principles for Teaching, Research, Knowledge Transfer, Consultancy and Related Activities.
- I have read and understand the University's policy and procedures on Safeguarding and Prevent.
- I undertake to abide by the ethical principles underlying the Declaration of Helsinki and the University Code of Conduct for Research, together with the codes of practice laid down by any relevant professional or learned society.
- If the activity is approved, I undertake to adhere to the study plan, the terms of the full application of which the Ethics Review Panel has given a favourable opinion and any conditions of the Ethics Review Panel in giving its favourable opinion.
- I undertake to seek an ethical opinion from the Ethics Review Panel before implementing substantial amendments to the study plan or to the terms of the full application of which the Ethics Review Panel has given a favourable opinion.
- I understand that I am responsible for monitoring the research at all times.
- If there are any serious adverse events, I understand that I am responsible for immediately stopping the research and alerting the Ethics Review Panel within 24 hours of the occurrence, via EthicsInfo@uclan.ac.uk.
- I am aware of my responsibility to be up to date and comply with the requirements of the law and relevant guidelines relating to security and confidentiality of personal data.
- I understand that research records/data may be subject to inspection for audit purposes if required in future.
- I understand that personal data about me as a researcher in this application is required by the Ethics, Integrity Unit and Governance Unit within Research and Enterprise Services, on behalf of the University, for an ethics review, and to evidence that the appropriate level of ethics review has been undertaken. Such data will be stored and managed in accordance with the principles established in the General Data Protection Regulations (GDPR)

and the Data Protection Act 2018.

- I understand that the information contained in this application, any supporting documentation and all correspondence with the Ethics Review relating to the application, will be subject to the provisions of the Freedom of Information Acts. The information may be disclosed in response to requests made under the Acts except where statutory exemptions apply.

- I understand that all conditions apply to any co-applicants and researchers involved in the study, and that it is my responsibility to ensure that they abide by them.

- **For Principal Investigator:** I understand my responsibilities to work within a set of ethical and other guidelines as set out by the University Policies and/or professional standards.

- **For Supervisor/Director of Studies:** I understand my responsibilities as Supervisor/Director of Studies, and will ensure, to the best of my abilities, that the student investigator abides by the University's Policy on Research Ethics at all times.

- **For the Student Investigator**: I understand my responsibilities to work within a set of ethical and other guidelines as agreed in advance with my Supervisor/Director of Studies and understand that I must comply with the University's regulations and any other applicable code of ethics at all times.

Please indicate below if you consent to your University Ethics Checklist, Ethics Application and other documentation being shared for training and review purposes. All forms and documents will be anonymised.

☐ Yes ☒ No

| | |
|---|---|
| ☒ **Signature of Principal Investigator: or**<br>☐ **Supervisor or Director of Studies** | 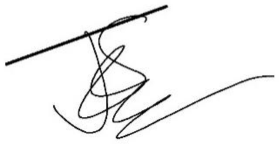 |
| **Print Name:** | DR JONATHAN SINCLAIR |
| **Date:** | 01/10/2023 |
| | |
| **Signature of Student Investigator:** | |
| **Print Name:** | |
| **Date:** | Click here to enter a date. |

Please submit the completed Ethics Form and Supporting Documentation to EthicsInfo@uclan.ac.uk
