# Supplementary material for: Effects of peppermint (Mentha x piperita L.) oil on cardiometabolic outcomes in patients with pre- and stage 1 hypertension: A placebo randomized controlled trial
Source: PLoS One. 2026 Apr 23;21(4):e0344538. doi: 10.1371/journal.pone.0344538 (PMC13105356; doi:10.1371/journal.pone.0344538)
Supplement: S3 File — (PDF) [file pone.0344538.s003.pdf]

University of Central Lancashire
Preston PR1 2HE
01772 201201
uclan.ac.uk

01 December 2023

Jonathan Sinclair
School of Health, Social Work and Sport
University of Central Lancashire

Dear Jonathan

Re: Health Ethics Review Panel Application
Unique Reference Number: HEALTH 01074

The Health Ethics Review Panel has granted approval of your proposal application 'Effects of oral peppermint supplementation on cardiometabolic parameters in participants with pre and stage 1 hypertension'. Approval is granted up to the end of project date.

It is your responsibility to ensure that:

- the project is carried out in line with the information provided in the forms you have submitted
- you regularly re-consider the ethical issues that may be raised in generating and analysing your data
- any proposed amendments/changes to the project are raised with, and approved by, the Ethics Review Panel
- you notify EthicsInfo@uclan.ac.uk if the end date changes or the project does not start
- serious adverse events that occur from the project are reported to the Ethics Review Panel
- a closure report is submitted to complete the ethics governance procedures (existing paperwork can be used for this purpose e.g. funder's end of grant report; abstract for student award or NRES final report. If none of these are available, use the e-Ethics Closure Report pro forma).

Yours sincerely

Lucy Hives
Deputy Vice-Chair
Health Ethics Review Panel

NB - Ethical approval is contingent on any health and safety checklists having been completed and necessary approvals gained as a result.
